# Dynamics of immune markers in different variants of post-psychotic depression after first-episode psychosis in young adult age

**DOI:** 10.1192/j.eurpsy.2022.931

**Published:** 2022-09-01

**Authors:** S. Zozulya, D. Tikhonov, V. Kaleda, T. Klyushnik

**Affiliations:** 1 Mental Health Research Centre, Laboratory Of Neuroimmunology, Moscow, Russian Federation; 2 Mental Health Research Centre, Department Of Youth Psychatry, Moscow, Russian Federation

**Keywords:** first-episode psychosis, post-psychotic depression, remission, immune markers

## Abstract

**Introduction:**

Research in recent decades focuses on understanding the role of the immune system in First-Episode Psychosis (FEP) at a young age. Our studies indicate that different stages of schizophrenia differ in the spectrum of inflammation markers. These indicators reflect the activity of the pathological process, using them as markers of the clinical state of patients at different stages of the disease.

**Objectives:**

To assess the relationship of immune markers with the clinical features of remission in patients after FEP.

**Methods:**

Fifty patients aged 15-25 years with post-psychotic depression (PD) after FEP (F20, F25) and 30 healthy men were included in the study. The follow-up period was two years. PD typological variants with positive affectivity (PA) (n=30) and negative affectivity (NA) (n=20) were distinguished. Leukocyte elastase (LE), a1-proteinase inhibitor (a1-PI) activity, and S-100B autoantibodies in plasma samples were measured.

**Results:**

The increase of LE and a1-PI activity in plasma of both types of PD patients compared to controls was detected (p<0.01). There was the highest LE activity and S-100B autoantibodies in PD with NA (p<0.05). The different dynamics of immune markers in both groups were correlated to the clinical features of remission. PD with PA was associated with a decrease in inflammatory markers (p<0.05) and a favorable prognosis. PD patients with NA had a further increase in LE activity and S-100B autoantibodies (p<0.01), and an unfavorable prognosis.

**Conclusions:**

The results confirm using the immune indicators as markers to assess the quality of remission after FEP in young adult age and the risk of recurrent psychotic attacks.

**Disclosure:**

No significant relationships.

